# Interaction between obesity and asthma in children and adolescents with hypertension based on NHANES 2007–2020

**DOI:** 10.3389/fpubh.2025.1526832

**Published:** 2025-04-28

**Authors:** Yizhe Ma, Luchun Wang, Mingyan Tao, Zhidan Bao, Renqiang Yu, Guihua Liu, Jing Liu, Hu Li

**Affiliations:** ^1^Department of Pediatrics, Jiangyin People’s Hospital of Nantong University, Jiangyin, China; ^2^Department of Neonatology, Affiliated Women’s Hospital of Jiangnan University, Wuxi Maternity and Child Health Care Hospital, Wuxi, China

**Keywords:** asthma, obesity, hypertension, children, NHANES

## Abstract

**Objectives:**

This study aims to elucidate the relationship between obesity and asthma in children and adolescents with hypertension based on the 2007–2020 National Health and Nutrition Examination Survey (NHANES).

**Methods:**

Weighted logistic regression models assessed obesity and asthma’s effects on hypertension risk, reported as odds ratio (OR) and 95% confidence interval (CI).

**Results:**

A cross-sectional analysis of 10,838 NHANES individuals aged 8–17 found 630 (5.8%) had hypertension, with significant differences in age, sex, poverty-to-income ratio, low birth weight, presence or absence of obesity and asthma, triglyceride concentration, fasting glucose concentration, and tobacco exposure compared to those without hypertension (*p* < .05). After adjusting for all covariates, children and adolescents with obesity or asthma had a higher risk of hypertension (obesity: adjusted OR 2.14, 95% CI 1.7–2.7; asthma: adjusted OR 1.43, 95% CI 1.1–1.9). Individuals with both obesity and asthma showed a significantly higher risk of hypertension compared to those without these conditions (adjusted OR 2.87, 95% CI 1.9–4.4) (*p* < .001). The synergistic effect of childhood obesity and asthma on hypertension risk remained robust after the stratified subgroup analysis based on age, sex, birth weight, and tobacco exposure.

**Conclusion:**

This study demonstrates that obesity and asthma exert a synergistic effect on childhood hypertension, emphasizing the need for comprehensive interventions targeting weight management and asthma control. There is a pressing need for future research to delve deeper into the mechanisms underlying the interactions between childhood asthma, obesity, and hypertension.

## Introduction

Arterial hypertension is recognized as the most important modifiable risk factor for all-cause morbidity and mortality worldwide, and it remains the most prevalent risk factor for cardiovascular disease (1). Hypertension in children and adolescents is a growing health problem with an increasing trend that is often overlooked (2). Hypertension in childhood or adolescence frequently progresses to hypertension in adulthood, and it is associated with detrimental outcomes, such as cardiovascular events, in adulthood (3). Hypertension in childhood and adolescence is also a risk factor for lower cognition during adulthood (4). Hypertension may affect the brain during early life, with a previous study showing that hypertension in youths is associated with lower performance on neurocognitive testing (5). A significant association has also been observed between hypertension in adolescence and adverse pregnancy outcomes in the National Family Health Survey in India (6). Therefore, the early identification and management of hypertension are crucial to prevent long-term cardiovascular complications that can arise from untreated high blood pressure. Comprehending the risk factors linked to hypertension in children is crucial for creating effective prevention and intervention measures.

Several factors contribute to hypertension in children and adolescents (7). Among them, childhood obesity and asthma are common pediatric health concerns that have garnered significant attention owing to their increasing prevalence and profound impact on the health and quality of life of children (8, 9). Childhood obesity is a risk factor for pediatric hypertension and asthma (10). The incidence of obesity among children has increased dramatically over the past few decades, with pooled estimates of 14.8% (95% CI 14.5–15.1%) and 22.2% (95% CI 21.6–22.8%) for children classified as overweight or carrying excess weight, respectively, leading to a heightened risk of comorbidity (11). Asthma and hypertension are complex conditions that frequently coincide in the adult population (12). A recent survey reported that pediatric asthma significantly increased the risk of hypertension in adulthood (13). However, the relationship between asthma and hypertension in children and adolescents is still unknown, and the interplay between these conditions, and the influence of obesity, remains inadequately explored.

This comprehensive cross-sectional study was performed to elucidate the associations between obesity and asthma as significant risk factors for hypertension in children and adolescents, while also exploring the potential synergistic effects of these conditions. This study aims to provide a clearer understanding of how these factors interact to influence hypertension risk. The findings of this research are anticipated to provide valuable insights into pediatric health, informing future interventions and public health strategies to mitigate hypertension in vulnerable populations.

## Methods

### Study population

**Figure 1:**
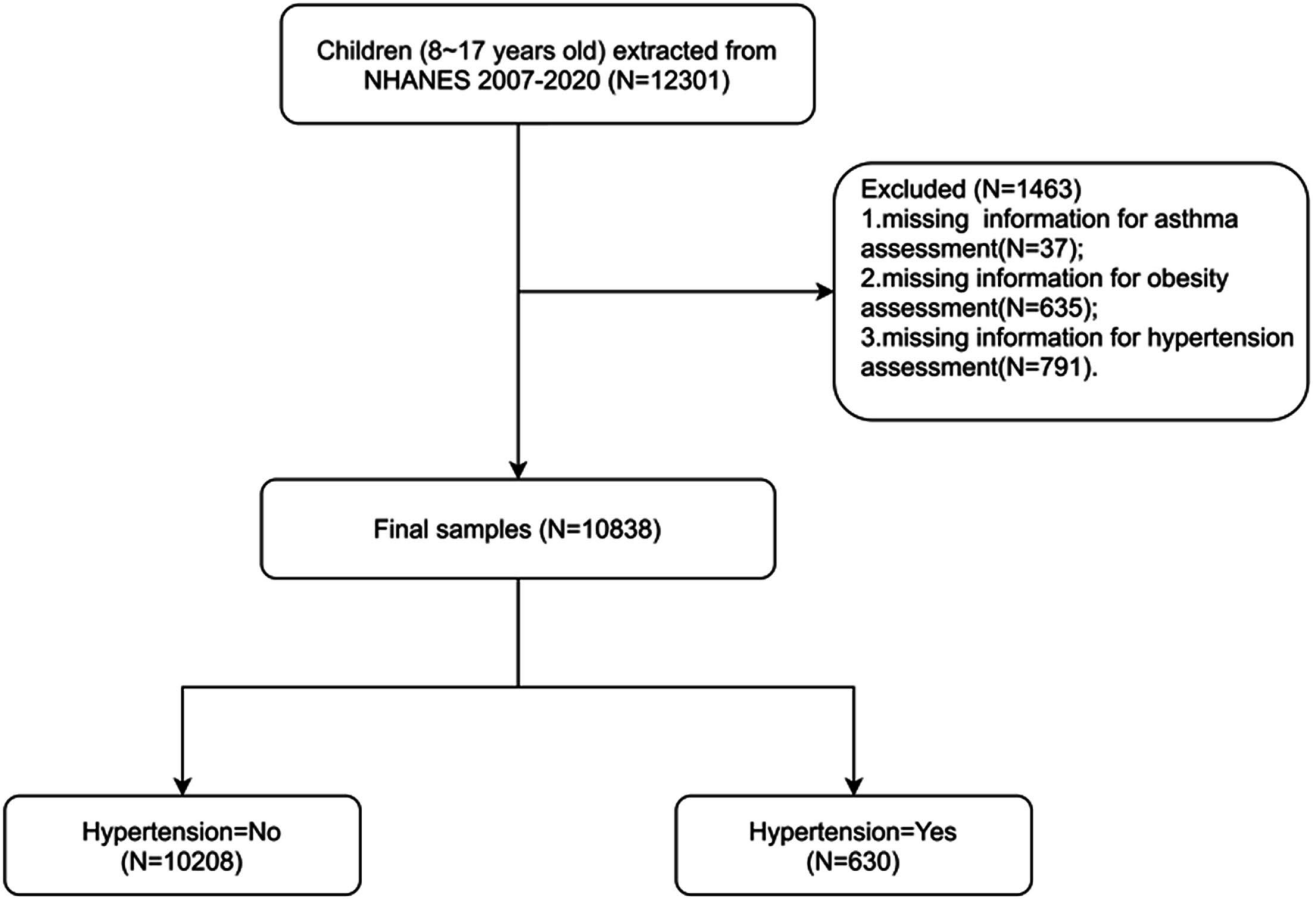
Flowchart of participants selection.

The National Health and Nutrition Examination Survey (NHANES) is a program of studies aimed at evaluating the health and nutritional condition of both adults and children across the United States.^1^ We used data from the 2007–2020 NHANES cycles, during which informed consent was obtained from all subjects with exemption from ethical approval. The NHANES gathers demographic information along with a comprehensive range of health-related data through household visits, screening evaluations, and laboratory tests conducted at a mobile examination facility as previously reported (14). This study included individuals from the NHANES who (1) were aged 8–17 years and (2) had complete assessment data for obesity, asthma, and hypertension. Children without key data, such as data on age and body mass index (BMI), were excluded. Finally, 10,838 individuals were enrolled (Figure 1).

### Exposures and outcomes

Hypertension was self-reported and blood pressure was measured by the NHANES standardized procedure, consistent with the previous literature (14). Participants were classified as having hypertension if they responded affirmatively to any of the subsequent inquiries as previously reported (14): BPQ020 – “Ever told you had high blood pressure?”; BPQ040A – “Taking prescription for hypertension?” The drug IDs included 41, 43, 44, 47, 48, 49, 53, 55, 56, and 342 as specified in DX_DRUG. Blood pressure measurements were conducted by qualified staff or medical practitioners, with all assessments carried out at designated mobile testing facilities. Three seated blood pressure measurements were taken for each participant and we computed the average of these readings for each participant. Hypertension in children and adolescents was diagnosed according to the 2017 Clinical Practice Guideline by the American Academy of Pediatrics (15). Obesity was assessed according to the BMI categories defined in the growth chart published by the Centers for Disease Control (BMI-for-age, 2–20 years, by sex and age).^2^ BMI ≥ 95th percentile was considered to indicate obesity. Asthma was assessed according to the following questions: MCQ010 – “Ever told you have asthma?”; MCQ035 – “Still have asthma?” as previously reported (16).

### Covariates and data collection

Data on sociodemographic variables, hematological indicators, and complications were collected. Sociodemographic variables included age, sex, race/ethnicity (Mexican–American, non-Hispanic Black, non-Hispanic White, other), BMI, birth weight, time per week play or exercise hard, sedentary time, hay fever, poverty-to-income ratio (PIR), and healthy eating index (HEI). Hematological indicators included cotinine level; homeostatic model assessment of insulin resistance (HOMA-IR) index; and total cholesterol, triglyceride (TAG), high-density lipoprotein cholesterol (HDL-C), low-density lipoprotein cholesterol (LDL-C), and fasting glucose concentrations in the serum. Low birth weight was defined as the birth weight <2,500 g. As previously reported, ideal physical activity was defined as an average of 60 min of moderate to vigorous physical activity daily for children and adolescents. Sedentary time was assessed by the NHANES questionnaires through the individual’s daily hours of TV, video, or computer use and was divided into three categories: < 3, 3–6, and ≥ 6 h (17). Serum cotinine levels of ≥.05 ng/mL were used to indicate tobacco exposure (18). Abnormal serum TC, HDL-C, LDL-C, fasting TAG and fasting glucose concentrations, and abnormal HOMA-IR index, were defined as follows: TC ≥ 200 mg/dL, TAG≥ 150 mg/dL, LDL-C ≥ 130 mg/dL, HDL-C ≤ 35 mg/dL, fasting glucose≥ 100 mg/dL and HOMA-IR ≥ 4.4 as prior literature (19).

### Statistical analysis

Subjects were categorized into two distinct cohorts based on their hypertension diagnosis: one group with hypertension and the other devoid of hypertension. We analyzed all the data using survey weights, strata, and primary sampling units provided by the National Center for Health Statistics consistent with previous literatures (16). Quantitative variables, such as age, were characterized using means and standard errors (SE). Inter-group comparisons were executed utilizing the T-test. For categorical data, both the count (n) and percentage (%) were computed, while the differences between groups were analyzed through the Chi-square (χ^2^) test. A weighted univariate logistic association analysis was performed for each potential confounding variable with hypertension as the outcome. All variables with statistically significant differences were further incorporated into the weighted multifactor model, and the potential confounding factors were screened out with *p* < .05 as the standard and identified as covariates. With *p* < .05 as the standard, a weighted multivariate logistic regression model was constructed to assess the association between obesity, asthma, and hypertension risk. The crude Model 1 did not adjust any confounders. Demographic factors including age, sex and race were adjusted in Model 2. In addition to demographic factors adjusted by Model 2, variables including PIR, TAG, HDL, low birth weight, sedentary time, fasting glucose, and tobacco exposure significantly linked to hypertension were adjusted in Model 3. All statistical evaluations were conducted using R version 4.3.3. Statistical significance was defined as a two-tailed *p*-value <.05.

## Results

### Baseline and demographic characteristics of the study population

Overall, the data of 10,838 individuals were analyzed. Among the participants, 630 (5.8%) had hypertension, while 10,208 (93.3%) did not. The characteristics of the participants are detailed in Table 1. The mean age was 12.6 ± 0.036 years. The sex distribution among the participants was comparable. The most common race/ethnicity was non-Hispanic White (*n* = 3,123 [55.1%]), followed by other (*n* = 2,647 [16.5%]), Mexican–American (*n* = 2,360 [14.7%]), and non-Hispanic Black (*n* = 2,708 [13.8%]). Approximately 22.4% of the participants had obesity, while 11.4% had asthma.

**Table 1:** Baseline and demographic characteristics of the included participants from the NHANES 2007–2020 cycles.

Variables	Total (*N* = 10,838)	Hypertension = No (*N* = 10,208)	Hypertension = Yes (*N* = 630)	Statistics	*P*
Age, Mean ± S.E	12.69 ± 0.04	12.59 ± 0.04	12.97 ± 0.15	*t* = 2.50	.014
Gender, n (%)				*χ*^2^ = 32.39	<.001
Male	5,492 (50.18)	5,066 (49.26)	426 (64.99)		
Female	5,346 (49.82)	5,142 (50.74)	204 (35.01)		
Race, n (%)				*χ*^2^ = 1.18	.317
Mexican American	2,360 (14.65)	2,236 (14.72)	124 (13.53)		
Non-Hispanic White	3,123 (55.05)	2,929 (54.96)	194 (56.42)		
Non-Hispanic Black	2,708 (13.83)	2,538 (13.72)	170 (15.64)		
Other Race	2,647 (16.47)	2,505 (16.60)	142 (14.41)		
Obesity, n (%)				*χ*^2^ = 49.00	<.001
No	8,173 (77.61)	7,808 (78.59)	365 (61.75)		
Yes	2,665 (22.39)	2,400 (21.41)	265 (38.25)		
Asthma, n (%)				*χ*^2^ = 8.34	.005
No	9,532 (88.61)	9,009 (88.91)	523 (83.91)		
Yes	1,306 (11.39)	1,199 (11.10)	107 (16.09)		
PIR, n (%)				*χ*^2^ = 3.90	.024
<1	3,067 (19.74)	2,859 (19.45)	208 (24.39)		
≥1	6,891 (73.73)	6,520 (74.04)	371 (68.71)		
Unknown	880 (6.54)	829 (6.51)	51 (6.90)		
PA, n (%)				*χ*^2^ = 0.86	.423
Not ideal physical activity	3,742 (34.50)	3,513 (34.28)	229 (38.03)		
Ideal physical activity	6,842 (63.49)	6,457 (63.70)	385 (60.06)		
Unknown	254 (2.01)	238 (2.02)	16 (1.91)		
HEI, n (%)				*χ*^2^ = 2.09	.128
<45	5,020 (47.68)	4,706 (47.42)	314 (51.83)		
≥45	5,020 (45.79)	4,749 (46.11)	271 (40.48)		
Unknown	798 (6.54)	753 (6.47)	45 (7.69)		
TC, mg/dl, n (%)				*χ*^2^ = 0.17	.801
<200	8,575 (79.31)	8,087 (79.38)	488 (78.19)		
≥200	648 (5.81)	599 (5.80)	49 (5.87)		
Unknown	1,615 (14.88)	1,522 (14.82)	93 (15.94)		
TAG, mg/dl, n (%)				*χ*^2^ = 3.49	.033
<150	2,259 (23.83)	2,144 (23.96)	115 (21.65)		
≥150	149 (1.56)	128 (1.47)	21 (3.01)		
Unknown	8,430 (74.61)	7,936 (74.56)	494 (75.34)		
LDL, mg/dl, n (%)				*χ*^2^ = 0.13	.864
<130	2,274 (24.06)	2,150 (24.11)	124 (23.21)		
≥130	132 (1.30)	120 (1.29)	12 (1.44)		
Unknown	8,432 (74.64)	7,938 (74.60)	494 (75.34)		
HDL, mg/dl, n (%)				χ^2^ = 2.77	.069
>35	8,688 (80.08)	8,203 (80.31)	485 (76.32)		
≤35	535 (5.04)	483 (4.87)	52 (7.74)		
Unknown	1,615 (14.88)	1,522 (14.82)	93 (15.94)		
Low birth weight, n (%)				*χ*^2^ = 12.36	<.001
No	7,355 (67.08)	6,987 (67.82)	368 (55.14)		
Yes	1,180 (9.40)	1,103 (9.13)	77 (13.73)		
Unknown	2,303 (23.53)	2,118 (23.05)	185 (31.13)		
Sedentary time, h, n (%)				*χ*^2^ = 1.89	.134
<3	1,999 (19.25)	1,895 (19.46)	104 (15.89)		
3 ~ 6	2,636 (23.37)	2,496 (23.52)	140 (21.03)		
≥6	3,651 (32.15)	3,427 (31.94)	224 (35.55)		
Unknown	2,552 (25.23)	2,390 (25.09)	162 (27.53)		
Hay fever, n (%)				*χ*^2^ = 1.66	.194
No	5,600 (48.67)	5,260 (48.42)	340 (52.73)		
Yes	910 (9.30)	850 (9.26)	60 (9.87)		
Unknown	4,328 (42.03)	4,098 (42.32)	230 (37.39)		
HOMA IR, n (%)				*χ*^2^ = 1.32	.269
<4.39	1,821 (19.98)	1,733 (20.12)	88 (17.76)		
≥4.39	574 (5.31)	527 (5.21)	47 (6.85)		
Unknown	8,443 (74.71)	7,948 (74.67)	495 (75.40)		
Fasting glucose, mg/dl, n (%)				*χ*^2^ = 3.69	.027
<100	1,853 (19.64)	1,766 (19.88)	87 (15.83)		
≥100	627 (6.42)	574 (6.24)	53 (9.28)		
Unknown	8,358 (73.94)	7,868 (73.88)	490 (74.89)		
Cotinine level, ng/ml, n (%)				*χ*^2^ = 9.70	<.001
<0.05	5,337 (51.85)	5,085 (52.56)	252 (40.30)		
≥0.05	3,904 (33.51)	3,615 (32.79)	289 (45.03)		
Unknown	1,597 (14.65)	1,508 (14.64)	89 (14.67)		

HDL, high-density lipoprotein; HEI, healthy eating index; HOMA-IR, homeostatic model assessment of insulin resistance; LDL, low-density lipoprotein; NHANES, National Health and Nutrition Examination Survey; PA, physical activity; PIR, poverty-to-income ratio; S.E, standard error; TAG, Triglyceride; TC, total cholesterol. Low birth weight was defined as the birth weight < 2,500 g. Ideal physical activity was defined as an average of 60 min of moderate to vigorous physical activity daily for children and adolescents. PIR < 1 indicated the low-income group. PIR≥1 indicated the moderate-income group.

### Characteristics of the participants with and without hypertension

The distribution of age, sex, PIR, low birth weight, and the presence or absence of obesity and asthma differed significantly between the two groups (all *p* < .05). Regarding lipid and glucose metabolism, the TAG and fasting glucose concentrations differed significantly between those with and without hypertension (both *p* < .05). Notably, the prevalence of tobacco exposure was significantly different between the two groups (*p* < .001). There were no discernible differences between the groups in the distribution of race, physical activity, HEI, TC concentration, LDL-C concentration, sedentary time, hay fever, or HOMA-IR index.

Weighted univariate logistic regression analysis was used to evaluate the association between potential confounding variables and outcomes. The results revealed that age, sex, race, PIR, TAG, HDL-C, low birth weight, sedentary time, fasting glucose, and tobacco exposure were significantly linked to the presence of hypertension (Table 2). Consequently, these factors were defined as covariates.

**Table 2:** Risk factors associated with hypertension in children and adolescents.

Variables	Outcome/total	OR (95% CI)	*P*
Age	630/10,838	1.05 (1.01–1.09)	.015
Sex			
Male	426/5,492	Ref	
Female	204/5,346	0.52 (0.42–0.66)	<.001
Race			
Mexican American	124/2,360	Ref	
Non-Hispanic White	194/3,123	1.12 (0.83–1.51)	.467
Non-Hispanic Black	170/2,708	1.24 (0.92–1.68)	.159
Other Race	142/2,647	0.94 (0.66–1.35)	.751
PIR			
<1	208/3,067	Ref	
≥1	371/6,891	0.74 (0.61–0.89)	.002
Unknown	51/880	0.85 (0.56–1.27)	.428
PA			
Not ideal physical activity	229/3,742	Ref	
Ideal physical activity	385/6,842	0.85 (0.65–1.11)	.234
Unknown	16/254	0.85 (0.38–1.91)	.697
HEI			
<45	314/5,020	Ref	
≥45	271/5,020	0.80 (0.64–1.01)	.062
Unknown	45/798	1.09 (0.71–1.67)	.693
TC, mg/dl			
<200	488/8,575	Ref	
≥200	49/648	1.03 (0.72–1.46)	.878
Unknown	93/1,615	1.09 (0.76–1.57)	.633
TAG, mg/dl			
<150	115/2,259	Ref	
≥150	21/149	2.27 (1.25–4.11)	.007
Unknown	494/8,430	1.12 (0.87–1.45)	.389
LDL, mg/dl			
<130	124/2,274	Ref	
≥130	12/132	1.16 (0.58–2.33)	.674
Unknown	494/8,432	1.05 (0.82–1.34)	.698
HDL, mg/dl			
>35	485/8,688	Ref	
≤35	52/535	1.67 (1.14–2.45)	.009
Unknown	93/1,615	1.13 (0.79–1.62)	.496
Low birth weight			
No	368/7,355	Ref	
Yes	77/1,180	1.85 (1.28–2.68)	.001
Unknown	185/2,303	1.66 (1.33–2.07)	<.001
Sedentary time			
<3	104/1,999	Ref	
3 ~ 6	140/2,636	1.10 (0.80–1.51)	.575
≥6	224/3,651	1.36 (1.01–1.84)	.044
Unknown	162/2,552	1.35 (0.95–1.90)	.091
Hay fever			
No	340/5,600	Ref	
Yes	60/910	0.98 (0.68–1.40)	.906
Unknown	230/4,328	0.81 (0.64–1.04)	.092
HOMA IR			
<4.39	88/1,821	Ref	
≥4.39	47/574	1.49 (0.92–2.42)	.107
Unknown	495/8,443	1.14 (0.83–1.59)	.416
Fasting glucose, mg/dl			
<100	87/1,853	Ref	
≥100	53/627	1.87 (1.17–2.99)	.010
Unknown	490/8,358	1.27 (0.94–1.73)	.123
Cotinine level, ng/ml			
<0.05	252/5,337	Ref	
≥0.05	289/3,904	1.79 (1.37–2.34)	<.001
Unknown	89/1,597	1.31 (0.92–1.86)	.135

CI, confidence interval; HDL, high-density lipoprotein; HEI, healthy eating index; HOMA-IR, homeostatic model assessment of insulin resistance; LDL, low-density lipoprotein; OR, odds ratio; PA, physical activity; PIR, poverty-to-income ratio; TAG, triglyceride; TC, total cholesterol; Ref, reference.

### Association between obesity and hypertension

Obesity and asthma were identified as significant risk factors for hypertension. The weighted multivariate logistic regression model was constructed to assess the association between obesity, asthma and hypertension incidence, respectively. As shown in Table 3, in contrast to their counterparts without obesity, children with obesity had an elevated risk of hypertension (unadjusted Model 1: crude OR 2.28, 95% CI 1.8–2.9; Model 2 [adjusted for demographic factors]: OR 2.27, 95% CI 1.8–2.9). After adjustment of age, sex, race, PIR, TAG, HDL, low birth weight, fasting glucose, and tobacco exposure in Model 3, children with obesity had a higher risk of hypertension (OR 2.14, 95% CI 1.7–2.7) than those without obesity.

**Table 3:** Associations of obesity/asthma with hypertension.

Variables	Model 1		Model 2		Model 3	
	OR (95% CI)	*P*	OR (95% CI)	*P*	OR (95% CI)	*P*
Obesity						
No	Ref		Ref		Ref	
Yes	2.28 (1.79–2.89)	<.001	2.27 (1.79–2.89)	<.001	2.14 (1.69–2.72)	<.001
Asthma						
No	Ref		Ref		Ref	
Yes	1.54 (1.14–2.07)	.005	1.50 (1.11–2.03)	.009	1.43 (1.06–1.94)	.022

CI, confidence interval; OR, odds ratio; Ref, reference.

Multivariate logistic regression of obesity and asthma for people with hypertension (Model 1, unadjusted model; Model 2, adjustment for age and sex as well as race; Model 3, adjustment for age, sex, race, PIR, TAG, HDL, low birth weight, sedentary time, fasting glucose, tobacco exposure).

### Association between asthma and hypertension

Table 3 also shows that there was a notable association between asthma and an elevated risk of hypertension (unadjusted Model 1: OR 1.54, 95% CI 1.1–2.1; Model 2 [adjusted for demographic factors including age, sex and race]: OR 1.50, 95% CI 1.1–2.0). Upon adjusting for age, sex, race, PIR, TAG, HDL, low birth weight, fasting glucose, and tobacco exposure in Model 3, participants with asthma exhibited a higher risk of hypertension (OR 1.43, 95% CI 1.1–1.9) than those without asthma.

### Interaction between obesity and asthma in children with hypertension

**Figure 2:**
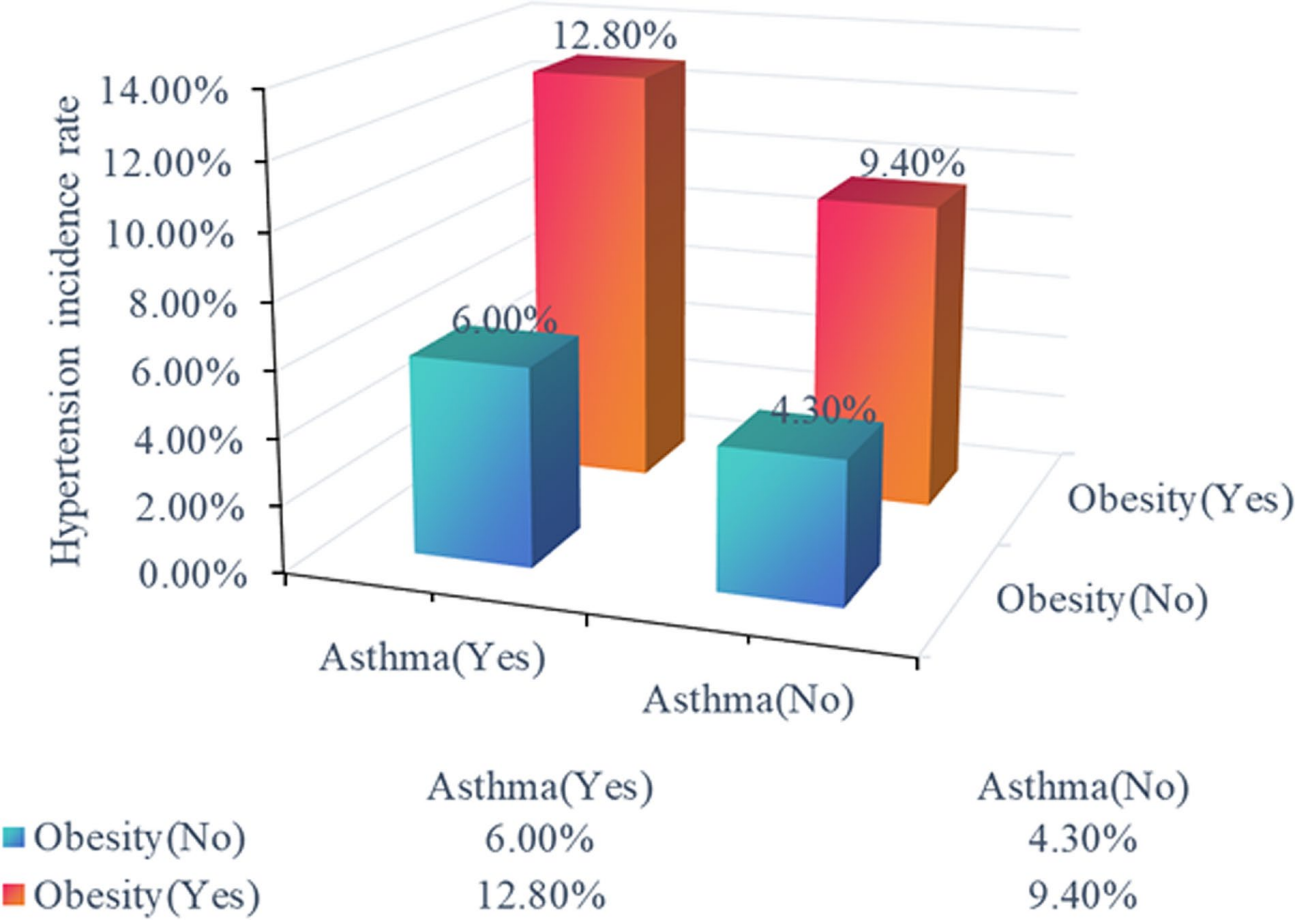
Hypertension risk across distinct cohorts. The distinct cohorts were shown as non-obese individuals without asthma, non-obese individuals with asthma, obese individuals without asthma, and obese individuals with asthma.

Furthermore, we explored the interplay between obesity and asthma in children with hypertension. We first analyzed hypertension risk across four distinct cohorts: individuals without obesity or asthma, non-obese individuals with asthma, obese individuals without asthma, and individuals with obesity and asthma. The inter-group comparisons revealed statistically significant differences in the incidence of hypertension across the four cohorts (*P*_trend_ < .001), with the group characterized by both obesity and asthma ranking the highest hypertension risk (Figure 2). There was also a significant synergistic effect of obesity and asthma on the risk of developing hypertension analyzed by the weighted multivariate logistic regression model (*P*_trend_ < .001 in all models, Table 4). Individuals with obesity and asthma exhibited a significantly increased risk of hypertension (OR 2.88, 95% CI 1.9–4.4) compared to those without conditions in Model 3 which adjusted for age, sex, race, PIR, TAG, HDL, low birth weight, fasting glucose, and tobacco exposure.

**Table 4:** Interaction between obesity and asthma with hypertension.

Variables	Model 1		Model 2		Model 3	
	OR (95% CI)	*P*	OR (95% CI)	*P*	OR (95% CI)	*P*
Non-obesity & Non-asthma	Ref		Ref		Ref	
Non-obesity & Asthma	1.47 (0.95–2.27)	.084	1.43 (0.92–2.23)	.108	1.37 (0.89–2.12)	.150
Obesity & Non-asthma	2.25 (1.74–2.92)	<.001	2.25 (1.73–2.93)	<.001	2.12 (1.64–2.74)	<.001
Obesity & Asthma	3.18 (2.14–4.72)	<.001	3.12 (2.08–4.67)	<.001	2.88 (1.89–4.37)	<.001
*P* for trend	<.001		<.001		<.001	

CI, confidence interval; OR, odds ratio; Ref, reference.

Multivariate logistic regression of obesity and asthma for people with hypertension (Model 1, unadjusted model; Model 2, adjustment for age and sex as well as race; Model 3, adjustment for age, sex, race, PIR, TAG, HDL, low birth weight, sedentary time, fasting glucose, tobacco exposure). The sample sizes for each subgroup are as follows: Non-obesity & Non-asthma: *n* = 7,288, with 312 hypertensive participants; Non-obesity & Asthma: *n* = 885, with 53 hypertensive participants; Obesity & Non-asthma: *n* = 2,244, with 211 hypertensive participants; Obesity & Asthma: *n* = 421, with 54 hypertensive participants.

Furthermore, to investigate the dynamics between obesity and asthma in relation to hypertension risk across diverse subgroups, we categorized the subjects into distinct subgroups by age, sex, birth weight, and tobacco exposure. Subgroup analyses were conducted within these subgroups. The findings revealed that, regardless of age, sex, birth weight, or tobacco exposure, the presence of obesity and asthma still increased the risk of hypertension (Table 5, *p* < .05 in Model 3).

**Table 5:** Interaction between obesity and asthma in children with hypertension in different subgroups.

Group	Variables	Model 1		Model 2		Model 3	
		OR (95%CI)	*P*	OR (95%CI)	*P*	OR (95%CI)	*P*
Age							
8 ~ 11 (281/4399)	Non-obesity & Non-asthma	Ref		Ref		Ref	
	Non-obesity & Asthma	1.45 (0.87–2.40)	.149	1.41 (0.85–2.32)	.182	1.38 (0.83–2.29)	.214
	Obesity & Non-asthma	1.70 (1.18–2.44)	.005	1.67 (1.15–2.42)	.008	1.67 (1.15–2.43)	.008
	Obesity & Asthma	2.45 (1.41–4.28)	.002	2.39 (1.33–4.27)	.004	2.20 (1.21–4.01)	.010
*P* for trend		<.001		<.001		<.001	
12 ~ 17 (349/5905)	Non-obesity & Non-asthma	Ref		Ref		Ref	
	Non-obesity & Asthma	1.48 (0.85–2.60)	.168	1.45 (0.82–2.57)	.203	1.39 (0.78–2.48)	.260
	Obesity & Non-asthma	2.66 (1.91–3.71)	<.001	2.69 (1.92–3.77)	<.001	2.62 (1.87–3.67)	<.001
	Obesity & Asthma	3.67 (2.22–6.08)	<.001	3.52 (2.10–5.91)	<.001	3.53 (2.07–5.99)	<.001
*P* for trend		<.001		<.001		<.001	
Sex							
Male (426/5492)	Non-obesity & Non-asthma	Ref		Ref		Ref	
	Non-obesity & Asthma	1.55 (0.95–2.54)	.082	1.55 (0.94–2.56)	.086	1.50 (0.92–2.45)	.105
	Obesity & Non-asthma	2.27 (1.65–3.13)	<.001	2.32 (1.67–3.20)	<.001	2.17 (1.56–3.01)	<.001
	Obesity & Asthma	3.33 (2.09–5.30)	<.001	3.41 (2.14–5.41)	<.001	3.41 (2.14–5.46)	<.001
*P* for trend		<.001		<.001		<.001	
Female (204/5346)	Non-obesity & Non-asthma	Ref		Ref		Ref	
	Non-obesity & Asthma	1.28 (0.60–2.74)	.517	1.27 (0.60–2.71)	.533	1.21 (0.60–2.43)	.597
	Obesity & Non-asthma	2.12 (1.30–3.46)	.003	2.16 (1.31–3.56)	.003	2.08 (1.27–3.41)	.004
	Obesity & Asthma	2.66 (1.22–5.84)	.015	2.90 (1.21–6.03)	.016	2.46 (1.08–5.62)	.033
*P* for trend		.002		.002		.003	
Low birth weight							
No (368/7355)	Non-obesity & Non-asthma	Ref		Ref		Ref	
	Non-obesity & Asthma	1.19 (0.69–2.06)	.520	1.16 (0.68–1.99)	.585	1.14 (0.66–1.96)	.632
	Obesity & Non-asthma	1.95 (1.43–2.67)	<.001	1.95 (1.42–2.67)	<.001	1.75 (1.29–2.38)	.001
	Obesity & Asthma	3.34 (2.12–5.29)	<.001	3.19 (1.99–5.13)	<.001	2.88 (1.78–4.67)	<.001
*P* for trend		<.001		<.001		<.001	
Yes (77/1180)	Non-obesity & Non-asthma	Ref		Ref		Ref	
	Non-obesity & Asthma	1.83 (0.69–4.85)	.224	2.06 (0.74–5.72)	.165	2.78 (1.10–6.99)	.030
	Obesity & Non-asthma	2.00 (0.78–5.09)	.145	2.04 (0.82–5.11)	.125	2.50 (1.12–5.61)	.026
	Obesity & Asthma	1.76 (0.61–5.06)	.294	1.96 (0.64–5.98)	.236	2.80 (0.83–9.42)	.096
*P* for trend		.095		.073		.009	
Unknown (185/2303)	Non-obesity & Non-asthma	Ref		Ref		Ref	
	Non-obesity & Asthma	1.61 (0.83–3.13)	.156	1.70 (0.85–3.40)	.132	1.57 (0.77–3.20)	.207
	Obesity & Non-asthma	3.24 (2.13–4.93)	<.001	3.10 (1.97–4.88)	<.001	2.95 (1.85–4.71)	<.001
	Obesity & Asthma	4.05 (1.71–9.59)	.002	4.37 (1.83–10.43)	.001	4.29 (1.77–10.37)	.002
*P* for trend		<.001		<.001		<.001	
Tobacco exposure							
No (252/5337)	Non-obesity & Non-asthma	Ref		Ref		Ref	
	Non-obesity & Asthma	1.44 (0.77–2.68)	.254	1.45 (0.77–2.72)	.248	1.50 (0.83–2.73)	.176
	Obesity & Non-asthma	2.60 (1.72–3.94)	<.001	2.51 (1.63–3.87)	<.001	2.49 (1.65–3.76)	<.001
	Obesity & Asthma	5.83 (3.08–11.06)	<.001	5.42 (2.78–10.54)	<.001	5.85 (2.82–12.14)	<.001
*P* for trend		<.001		<.001		<.001	
Yes (289/3904)	Non-obesity & Non-asthma	Ref		Ref		Ref	
	Non-obesity & Asthma	1.46 (0.76–2.81)	.250	1.43 (0.74–2.77)	.287	1.36 (0.71–2.62)	.349
	Obesity & Non-asthma	1.64 (0.93–2.88)	.088	1.76 (1.00–3.08)	.048	1.69 (0.97–2.96)	.065
	Obesity & Asthma	1.81 (1.26–2.58)	.001	1.89 (1.31–2.74)	.001	1.83 (1.24–2.69)	.003
*P* for trend		.001		<.001		.001	
Unknown (89/1597)	Non-obesity & Non-asthma	Ref		Ref		Ref	
	Non-obesity & Asthma	1.13 (0.44–2.91)	.793	1.13 (0.45–2.79)	.798	1.11 (0.45–2.73)	.815
	Obesity & Non-asthma	2.35 (1.18–4.69)	.016	2.40 (1.26–4.60)	.009	2.39 (1.25–4.54)	.009
	Obesity & Asthma	2.92 (1.12–7.61)	.029	3.12 (1.23–7.91)	.017	2.82 (1.05–7.58)	.040
*P* for trend		.006		.002		.005	

CI, confidence interval; OR, odds ratio; Ref, reference.

Multivariate logistic regression of obesity and asthma for people with hypertension (Model 1, unadjusted model; Model 2, adjustment for age and sex as well as race; Model 3, adjustment for age, sex, race, PIR, TAG, HDL, low birth weight, sedentary time, fasting glucose, tobacco exposure). The sample sizes for each subgroup were featured by hypertensive cases/total cases.

## Discussion

In this study, we utilized nationally representative data of children aged 8–17 years derived from the 2007–2020 NHANES cycles to evaluate the multifaceted nature of hypertension in the pediatric population of the United States. The results revealed significant disparities in the characteristics between participants with and without hypertension, particularly in terms of age, sex, PIR, TAG, birth weight, fasting glucose, and tobacco exposure. The prevalence of hypertension in children and adolescents varies widely due to demographic factors such as age, sex, and geographic location (2). In a recent investigation, it was found that the occurrence of hypertension was 4.3% (95% CI 2.8–6.6%) in children who were 6 years, with the highest rate observed at 7.9% (95% CI 5.8–10.8%) in the population aged 14 years (2). Typically, boys exhibit higher rates of hypertension than girls during childhood, although this trend may reverse during adolescence due to hormonal changes and lifestyle factors (20). Children from poorer socioeconomic backgrounds are particularly vulnerable to hypertension, which may be due to their limited access to healthy foods, lack of physical activity, and higher stress levels (21). Furthermore, low birth weight is considered to be a risk factor for hypertension in adulthood (22). The serum TAG concentration and fasting glucose concentration are recognized markers of an adverse cardiovascular prognosis in patients with hypertension (23). Additionally, a sedentary lifestyle correlates with an increased prevalence of obesity and hypertension among the pediatric population (10). Childhood tobacco exposure is considered to be a risk factor for hypertension in children and is associated with adult preclinical atherosclerosis and lung diseases (24, 25). Consistent with the previous studies, our analysis also revealed that age, sex, race, PIR, TAG, HDL-C, low birth weight, sedentary time, fasting glucose concentration, and tobacco exposure were all risk factors for childhood hypertension.

Notably, obesity and asthma both emerged as pivotal risk factors for hypertension in children and adolescents in the present study. Individuals with obesity or asthma both exhibited a markedly elevated risk of hypertension, even after subsequent adjustment for the confounding variables. This aligns with the existing literature, highlighting the strong association between obesity and blood pressure elevation in children, suggesting that obesity may act as a precursor to hypertension, potentially through mechanisms such as increased vascular resistance and altered metabolic profiles (26). Moreover, the association between asthma and hypertension was also significant, indicating that asthma may contribute to hypertension risk. The well-accepted mechanisms driving this relationship may involve systemic inflammation associated with asthma, which can lead to vascular dysfunction and increased blood pressure (27). Additionally, the medications used to manage asthma, such as corticosteroids, have been linked to weight gain and other metabolic changes that could further exacerbate hypertension risk (27). Previous studies have reported the interaction between asthma and cardiovascular health in adults (28). The present study reported an association between asthma and hypertension in the pediatric population for the first time. Pediatric patients with asthma are more likely to have hypertension than those without asthma, and the association between these conditions may have been underestimated in children or adolescents previously. This may be an interplay of the combination of genetic factors, nervous system dysfunction, age, diet, and lifestyle, as well as systemic inflammation (12).

As previously reported, obesity and asthma feature a reciprocal interaction whereby each disease impacts the severity of the other (29). Interestingly, the present study showed that children with both obesity and asthma were nearly three times more likely to develop hypertension compared to those without these conditions. This outlined a synergistic effect of obesity and asthma on hypertension risk, despite the shared risk factors, including age, sex, low birth weight, and tobacco exposure. Both conditions share common inflammatory pathways that can disrupt cardiovascular health. Metabolic disorders further complicate this landscape, as it encompasses a range of risk factors, including insulin resistance, dyslipidemia, which can co-occur with asthma and obesity in pediatric populations. The interaction between neuroendocrine factors and immune responses is also crucial in recent studies (30–32). Additionally, lifestyle factors such as diet, physical activity, and socioeconomic status can significantly influence these conditions, necessitating a holistic approach to intervention (33). Understanding these interactions is vital for developing targeted interventions that address not only the individual conditions of asthma and obesity but also their combined effects on hypertension risk in children.

### Study strengths and limitations

The strength of this study lies in its ability to capture a wide array of demographic and health-related variables based on the NHANES data, allowing for a nuanced analysis of the specific contributions of obesity and asthma to hypertension risk. However, this study also has several limitations that should be considered. The study adopted a cross-sectional design, which complicates the confirmation of temporal sequences and causal relationships. The absence of longitudinal data also restricted our ability to infer the directionality of these associations. Obstructive sleep apnea (OSA) is also an increasing chronic respiratory disorder associated with obesity and asthma (34). The prevalence of hypertension was notably elevated among children experiencing more severe OSA (35). But in our study population from the NHANES 2007–2020 dataset, there is not enough information on OSA, which could be a significant confounder. Additionally, some of the disease conditions of asthma and hypertension were self-reported based on the NHANES data, we also lacked information on asthma control and medication usage, which further limited our analysis. Further longitudinal studies are essential to gain a deeper insight into the underlying pathophysiological mechanisms that govern the interactions among asthma, obesity, and hypertension.

## Conclusion

In conclusion, we identified a significant association between obesity and hypertension, underscoring the need for targeted interventions in children and adolescents who are categorized as overweight. Furthermore, the findings revealed a notable correlation between asthma and hypertension, suggesting that respiratory conditions may exacerbate cardiovascular risk in childhood. Importantly, the interaction between obesity and asthma presents a complex dynamic that warrants further investigation, as the two conditions exert synergistic effects on childhood hypertension.

## Data Availability

Publicly available datasets were analyzed in this study. This data can be found here: https://www.cdc.gov/nchs/nhanes/index.htm.

## Footnotes

^1^ https://www.cdc.gov/nchs/nhanes/index.htm

^2^ https://www.cdc.gov/growth-chart-training/hcp/computer-programs/sas.html
